# Acute-phase proteins and incidence of diabetes: a population-based cohort study

**DOI:** 10.1007/s00592-016-0903-8

**Published:** 2016-08-31

**Authors:** Iram Faqir Muhammad, Yan Borné, Bo Hedblad, Peter M. Nilsson, Margaretha Persson, Gunnar Engström

**Affiliations:** Department of Clinical Sciences, Lund University, CRC 60:13, Jan Waldenströms gata 35, 20502 Malmö, Sweden

**Keywords:** Acute-phase proteins, Diabetes, Incidence, Ceruloplasmin, Alpha-1-antitrypsin, Orosomucoid, Haptoglobin, CRP

## Abstract

**Aims:**

To examine the relationship between plasma levels of the acute-phase proteins ceruloplasmin, alpha-1-antitrypsin, orosomucoid, haptoglobin and C-reactive protein (CRP), and incidence of diabetes in the population-based Malmö Diet and Cancer Study—Cardiovascular Cohort (MDCS-CC).

**Methods:**

The study population consists of 4246 participants (aged 46–67 years, 60.8 % women) with no previous history of diabetes. Participants were followed, and incidence of diabetes was assessed by linkage with national registers and a clinical re-examination of the cohort. Cox proportional hazard regression analysis was used to compare incidence of diabetes in relation to sex-specific quartiles of the acute-phase proteins.

**Results:**

During a mean follow-up period of 15.6 ± 3.4 years, a total of 390 participants were diagnosed with diabetes. Orosomucoid, haptoglobin, and CRP showed a significant increased risk of diabetes after adjustment for potential confounders. However, further adjustments for fasting glucose at baseline resulted in significant association only for CRP. The multivariable-adjusted hazard ratios (HR: 4th vs. 1st quartile) were 1.18 (95 % CI: 0.83–1.67; *p* = 0.51), 1.19 (CI: 0.85–1.62; *p* = 0.10), and 1.40 (CI: 1.01–1.95; *p* = 0.046) for orosomucoid, haptoglobin, and CRP respectively.

**Conclusion:**

The study demonstrated that there are associations between orosomucoid, haptoglobin and CRP and the risk of incidence of diabetes. However, after additional adjustment for fasting glucose levels at baseline, the association stayed significant only for CRP.

## Introduction

Diabetes, a major non-communicable disease, is one of the foremost public health and development challenges faced by the world today. The global prevalence of diabetes in 2014 was estimated to be 9 % among adults older than 18 years [1]. The World Health Organization projects that diabetes will be the seventh leading cause of death in the world by 2030 [2]. Age, obesity, physical inactivity, and family history of the disease are the major risk factors for type 2 diabetes [3].

Chronic low-grade inflammation has also been associated with the incidence of diabetes [4]. The relationship between development of diabetes and various inflammatory proteins has been explored in several studies previously; however, the results have been inconsistent [5–9]. The Women’s Health Study, a prospective study about the health of middle-aged women in the USA found that elevated level of C-reactive protein (CRP) and interleukin-6 (IL-6) predict development of type 2 diabetes in women [7]. Plasminogen activator inhibitor-1 (PAI-1) but not fibrinogen and CRP were found to predict type 2 diabetes in the Insulin Resistance Atherosclerosis Study, IRAS [5]. The results from the National Health and Nutrition Examination Survey (NHANES) Epidemiologic Follow-up Study in the USA showed significant association of leukocyte count with diabetes incidence among women but not in men; however, erythrocyte sedimentation rate (ESR) was not associated with diabetes [8]. In another study, individuals at high risk of type 2 diabetes, using Hemoglobin A1c (HbA1_c_) criterion, showed an unfavorable inflammatory profile of five inflammatory markers (high-sensitivity hs-CRP, ESR, fibrinogen, white blood cell WBC count, and complement C3) as compared with control subjects [10].

Various acute-phase proteins have been associated with the development of diabetes and impaired glucose tolerance. Schmidt et al. studied the association of fibrinogen, orosomucoid, haptoglobin, and alpha-1-antitrypsin in new diabetes cases. The results showed strong association of orosomucoid with incidence of diabetes even when adjusted for different confounders [11]. Another study investigated the association of high levels of a score of five acute-phase proteins (fibrinogen, orosomucoid, alpha-1-antitrypsin, haptoglobin, and ceruloplasmin) with future weight gain [12]. Elevated levels of acute-phase proteins have been showed to associate with the prevalence of diabetes among overweight and obese men in the Malmö Preventive Project (MPP) [13].

The *aim* of this prospective study is to investigate the relationship between plasma levels of five acute-phase proteins (CRP, ceruloplasmin, alpha-1-antitrypsin, orosomucoid, and haptoglobin) at baseline and incidence of diabetes in a population-based cohort.

## Materials and methods

### Study population

The Malmö Diet and Cancer cohort (MDCS) included men born between 1923 and 1945 and women born between 1923 and 1950. Recruitment was carried out between March 1991 and September 1996. Out of 74,138 participants who were invited, a total of 30,447 participants (60 % women) attended the baseline examinations [14]. A sub-cohort was obtained from the MDCS by randomly selecting participants between October 1991 and February 1994, with a primary aim of studying the epidemiology of carotid artery disease. The sub-cohort consisted of 6103 (2572 men and 3531 women) participants. Out of these, 5540 subjects agreed to give fasting blood samples for laboratory analysis [15]. Participants lacking data on acute-phase proteins (*n* = 858) ceruloplasmin, alpha-1-antitrypsin, orosomucoid, and haptoglobin were excluded from the analysis. For the acute-phase protein CRP, 113 participants had missing data. Participants with history of diabetes (*n* = 192) at baseline and those with fasting whole blood glucose of ≥6.1 mmol/L (*n* = 359) were also excluded from the study, hence leaving a final study population of 4246 (see “Appendix” for flowchart).

### Baseline examinations

The MDCS baseline measurements comprised of a self-administered questionnaire, anthropometric measurements, and collection of blood samples [16]. Non-fasting blood samples were also drawn which were separated and stored in the biological bank at −80 °C. Information on family history of diabetes, current use of anti-hypertensive or anti-diabetic medications, smoking habits, leisure-time physical activity, educational level, and marital status were obtained from the questionnaire. Participants were classified into four categories regarding smoking habits: current smokers, occasional smokers, ex-smokers, and non-smokers. Low level of leisure-time physical activity was defined as the lowest quartile of a score revealed through 18 questions covering a range of activities in the four seasons [17]. The measurements of waist circumference (in cm) were carried out midway between the lowest rib margin and the iliac crest. Educational level was defined into three categories: school years <9, 9–12 and >12, respectively [14].

Information on fasting glucose was obtained through blood samples collected after an overnight fast. Participants with fasting whole blood glucose of ≥6.1 mmol/l, which corresponds to a fasting plasma glucose concentration of ≥7.0 mmol/l, as well as those who self-reported diabetes in the questionnaire or were taking anti-diabetic medication were classified as diabetics [18]. Blood glucose and HbA1_c_ were analyzed from fasting blood samples by standardized procedures at the Department of Clinical Chemistry, Malmö University hospital. HbA1_c_ was measured by ion exchange chromatography. The reference values in non-diabetic individuals were 3.9–5.3 % (19–34 mmol/mol). Insulin was measured by radioimmunoassay in mIU/l [15].

Measurements of fasting blood glucose and high-density lipoprotein (HDL) were performed in MDCS-CC on fresh blood samples according to standard procedures at the Department of Clinical Chemistry, University Hospital Malmö. Low-density lipoprotein cholesterol (LDL) concentration was calculated according to Friedewald’s formula.

High-sensitive CRP in plasma was analyzed using the Tina-quant^®^ CRP latex assay (Roche Diagnostics, Basel, Switzerland). Plasma levels of ceruloplasmin, orosomucoid, haptoglobin, and alpha-1-antitrypsin were analyzed using Cobas c-systems (Roche Diagnostics GmbH, Germany). The reference values were 0.15–0.30 g/l for men and 0.16–0.45 g/l for women for ceruloplasmin [19]. For alpha-1-antitrypsin, the reference value was 0.9–2.0 g/l [19]. The reference value for orosomucoid was 0.5–1.2 g/l [19]. Finally, the reference value for haptoglobin was 0.3–2.0 g/l (www.roche.com) [19].

### Follow-up and endpoint definition

All participants in the study were followed from the baseline examination until first diagnosis of diabetes, emigration from Sweden, death or December 31, 2009, whichever came first. New onset cases of diabetes in the cohort were gathered from different sources which have been described in detail previously [20]. To summarize, incident diabetes was identified by using the Malmö HbA1c register (MHR), the Swedish National Diabetes Register (NDR), the Swedish inpatient register, the Swedish outpatient register, the nationwide Swedish drug prescription register, and the regional Diabetes 2000 register of the Skåne region [20, 21]. The cases in the Diabetes 2000 register and NDR were based upon diagnosis by a physician according to recognized diagnostic criteria (fasting plasma glucose concentration of ≥7.0 mmol/l, measured on two different occasions) [18].

### Statistical analysis

CRP was log transformed due to the skewed distribution. The distribution for ceruloplasmin, haptoglobin, alpha-1-antitrypsin, and orosomucoid was approximately normal. Mean values (±standard deviations) and proportions were used to describe the baseline characteristics of the cohort for men and women. The acute-phase proteins were categorized into sex-specific quartiles, i.e., with equal proportion of men and women in each quartile. Linear regression was used to assess the association between acute-phase proteins and fasting blood glucose. Cox proportional hazard regression was used to compare the incidence of diabetes in different quartiles of individual proteins for the whole cohort. Hazard ratios (HRs) with 95 % confidence interval (CI) were calculated. The significance level was defined as a *p* value of less than 0.05.

**Fig. 1:**
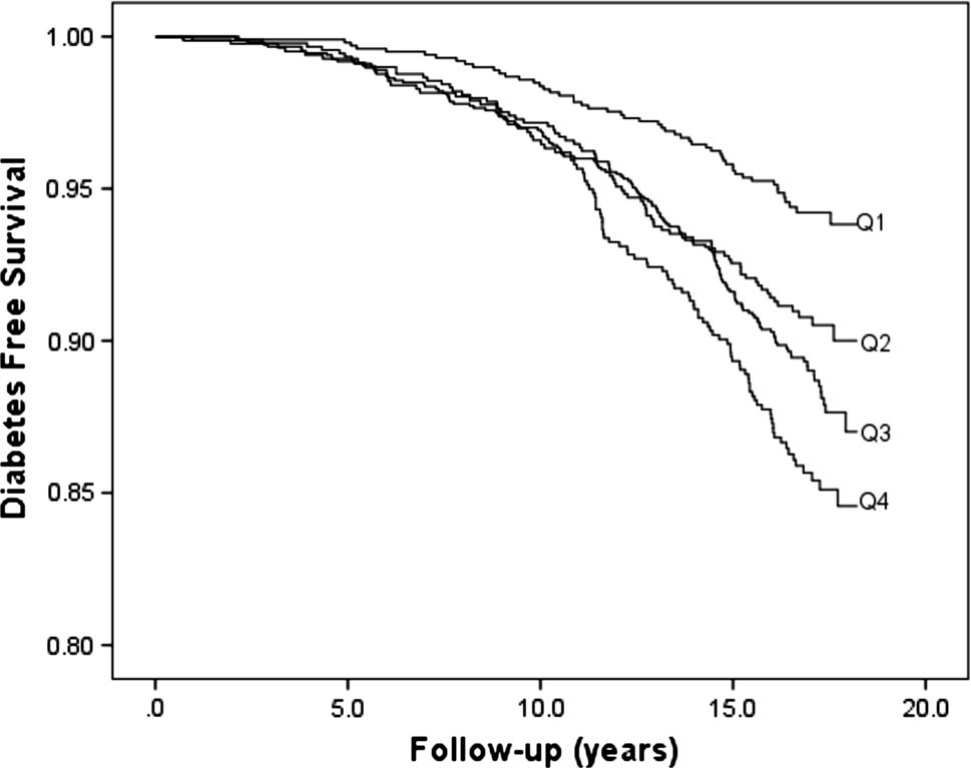
Diabetes-free survival in relation to sex-specific quartiles (Q1–Q4) of orosomucoid

**Fig. 2:**
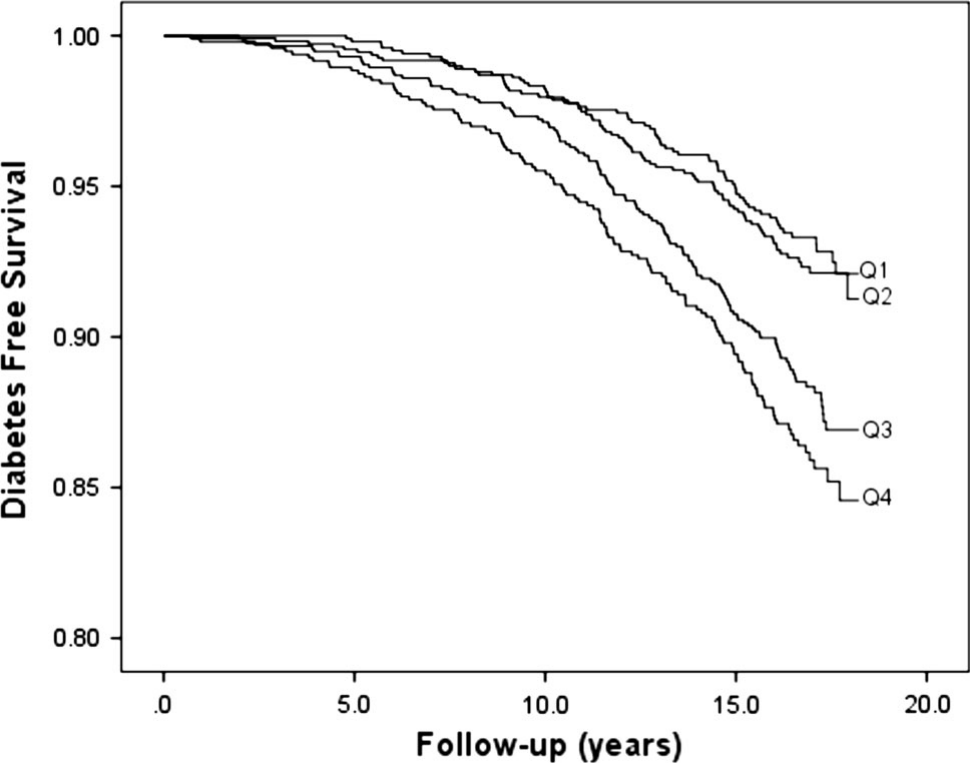
Diabetes-free survival in relation to sex-specific quartiles (Q1–Q4) of haptoglobin

**Fig. 3:**
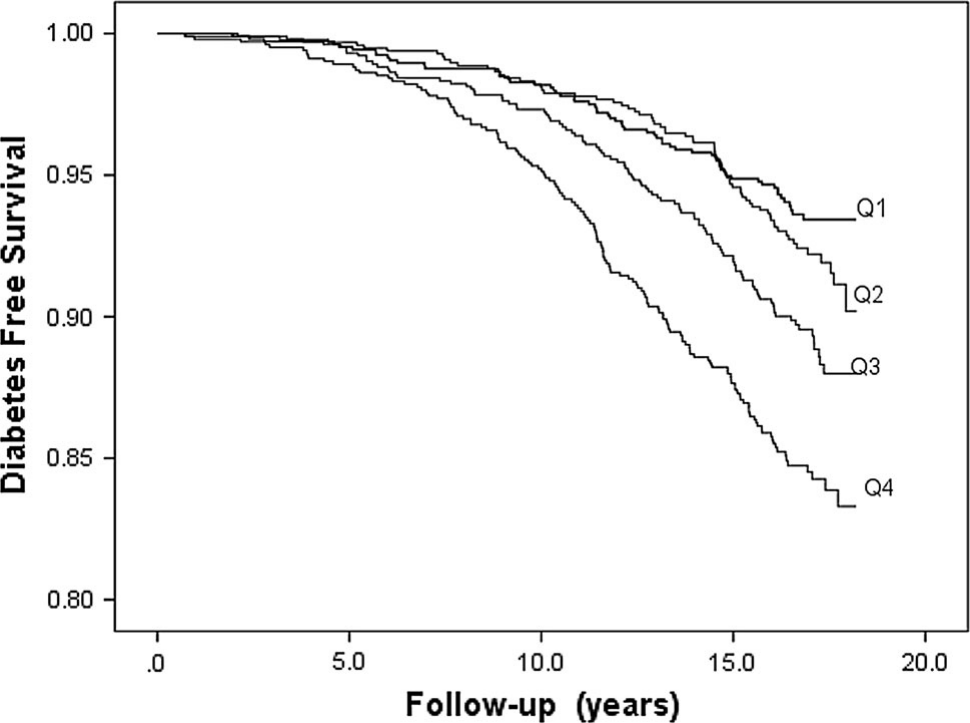
Diabetes-free survival in relation to sex-specific quartiles (Q1–Q4) of CRP

The HRs were adjusted for potential confounders in all the models. In the first model (Model 1), adjustments were made for age and sex. The second model (Model 2) was further adjusted for waist circumference, smoking habits (current and occasional smokers), physical activity, use of anti-hypertensive medication, systolic BP, HDL, and educational level. Finally, additional adjustments were done for baseline fasting blood glucose in Model 3 to demonstrate whether the associations were cross-sectional or prospective. Possible interactions between the plasma proteins and other risk factors for diabetes were investigated by introducing interaction terms in the multivariable model. The fit of the proportional hazards model was confirmed by plotting the incidence rate over time. The Kaplan–Meier curve was used to illustrate diabetes-free survival in relation to orosomucoid, haptoglobin, and CRP in sex-specific quartiles (Figs. 1, 2, 3).

Possible effect modification was assessed by modeling interaction terms between the acute-phase proteins and age, sex, and waist circumference, in the adjusted (Model 2) Cox’s proportional hazards models. All analyses were performed using IBM SPSS version 22 0 (SPSS Inc., Chicago, IL, USA).

Ethical approval was obtained from the Lund University Ethics Review Committee (LU51/90). All participants gave written informed consent.

## Results

### Baseline characteristics

The baseline characteristics of the study population in relation to sex have been presented in Table 1. Men and women were rather similar in terms of baseline characteristics. Men had slightly higher levels of glucose, waist circumference, and higher blood pressure and used more anti-hypertensive medications. Women had higher ceruloplasmin levels than men.

**Table 1:** Baseline characteristics of the MDCS-CC among men and women (*N* = 4246)

Baseline characteristics	Men	Women
*N*	1664	2582
Incidence of diabetes *n* (*n* per 1000 p-y)	181 (7.19)	209 (5.09)
Glucose (mmol/l)	5.01 ± 0.4	4.80 ± 0.4
Age at baseline (years)	57.63 ± 6.0	57.39 ± .5.9
Ceruloplasmin (mg/l)	0.46 ± 0.10	0.54 ± 0.12
Alpha-1-antitrypsin (mg/l)	1.19 ± 0.26	1.21 ± 0.26
Orosomucoid (mg/l)	0.71 ± 0.21	0.69 ± 0.20
Haptoglobin (mg/l)	1.30 ± 0.58	1.31 ± 0.51
CRP (mg/l) (GM [5th–95th percentiles])	1.35 (0.30–8.30)	1.29 (0.30–7.21)
Waist circumference (cm)	92.15 ± 9.4	76.18 ± 9.5
Low physical activity (%)	20.9	22.4
Current smoking status (%, current and occasional smokers)	26.1	25.6
Use of anti-hypertensive medication (%)	15.0	13.3
Systolic BP (mmHg)	142.4 ± 18.5	139.2 ± 18.9
HDL (mmol/l)	1.23 ± 0.30	1.53 ± 0.35
Low educational level (%)	46.4	43.3

Data are mean ± SD unless otherwise indicated

*p*-*y* person year

### Correlations between acute-phase proteins and glucose

Ceruloplasmin, orosomucoid, haptoglobin, and CRP showed significant and positive correlations with fasting glucose, after adjustment for age and sex as shown in Table 2. This association remained statistically significant for orosomucoid and haptoglobin after further adjustment for the other covariates.

**Table 2:** Correlations between acute-phase proteins and glucose in non-diabetic individuals

	Glucose (age, sex)	Glucose (risk factors)^a^
Ceruloplasmin	0.035**	0.007
Alpha-1-antitrypsin	−0.004	−0.004
Orosomucoid	0.152***	0.075***
Haptoglobin	0.146***	0.095***
LnCRP	0.105***	−0.001

Values are standardized beta coefficients, from a linear regression model with the acute-phase protein as the dependent variable

* *p* < 0.05; ** *p* < 0.01; *** *p* < 0.001

^a^Risk factors: adjusted for age, sex, waist circumference, smoking, use of anti-hypertensive medication, systolic BP, HDL, low educational level, and low physical activity

### Incidence of diabetes in relation to acute-phase proteins

The mean follow-up period was 15.6 ± 3.4 years. A total of 390 participants (181 men and 209 women) were diagnosed with diabetes during the follow-up period. The incidence of diabetes was 7.19 per 1000 person-years in men and 5.09 per 1000 person-years in women.

Of the five acute-phase proteins, subjects in the fourth compared to the first quartile of orosomucoid, haptoglobin and CRP, respectively, had significantly higher risk of incidence of diabetes in the first model (Table 3). Figures 1, 2, and 3 illustrate the diabetes-free survival by quartiles of these acute-phase proteins. After adjustment for covariates in Model 2, this risk increase remained for orosomucoid (HR 1.46; 95 % CI 1.03–2.08), haptoglobin (HR 1.49; 95 % CI 1.08–2.05), and CRP (HR 1.44; 95 % CI 1.03–2.00). However, after also taking fasting blood glucose into account in Model 3, only CRP remained significant (HR 1.40; 95 % CI 1.01–1.95; Table 3).

**Table 3:** Incidence of diabetes in relation to sex-specific quartiles of five acute-phase proteins

	Q1	Q2	Q3	Q4	*p* for trend
*Ceruloplasmin*					
Mean ± SD	0.39 ± 0.050	0.47 ± 0.048	0.52 ± 0.046	0.65 ± 0.112	
Incidence of diabetes *n* (*n*/1000)^a^	74 (4.4)	104 (6.3)	111 (6.9)	101 (6.0)	
Model 1	1	1.41 (1.05–1.90)	1.52 (1.13–2.04)	1.32 (0.98–1.79)	0.074
Model 2	1	1.29 (0.95–1.77)	1.33 (0.97–1.80)	1.09 (0.79–1.49)	0.739
Model 3	1	1.25 (0.92–1.71)	1.36 (1.00–1.85)	1.06 (0.78–1.46)	0.748
*Alpha*-*1*-*antitrypsin*					
Mean ± SD	0.88 ± 0.157	1.14 ± 0.049	1.27 ± 0.047	1.55 ± 0.197	
Incidence of diabetes *n* (*n*/1000)^a^	82 (5.3)	119 (5.6)	101 (6.7)	88 (6.1)	
Model 1	1	1.10 (0.83–1.46)	1.21 (0.90–1.62)	1.19 (0.88–1.61)	0.191
Model 2	1	1.20 (0.89–1.61)	1.20 (0.88–1.64)	1.18 (0.85–1.62)	0.374
Model 3	1	1.27 (0.94–1.71)	1.32 (0.97–1.81)	1.27 (0.91–1.75)	0.153
*Orosomucoid*					
Mean ± SD	0.46 ± 0.059	0.60 ± 0.000	0.74 ± 0.050	1.10 ± 0.163	
Incidence of diabetes *n* (*n*/1000)^a^	53 (3.3)	79 (5.5)	152 (6.6)	106 (8.4)	
Model 1	1	1.66 (1.17–2.35)	1.99 (1.45–2.71)	2.52 (1.81–3.50)	<0.001
Model 2	1	1.30 (0.91–1.87)	1.25 (0.90–1.74)	1.46 (1.03–2.08)	0.058
Model 3	1	1.16 (0.81–1.67)	1.06 (0.76–1.48)	1.18 (0.83–1.67)	0.510
*Haptoglobin*					
Mean ± SD	0.68 ± 0.182	1.07 ± 0.099	1.44 ± 0.110	2.05 ± 0.410	
Incidence of diabetes *n* (*n*/1000)^a^	66 (4.1)	79 (4.5)	126 (7.0)	119 (8.2)	
Model 1	1	1.07 (0.77–1.48)	1.70 (1.26–2.29)	2.06 (1.52–2.78)	<0.001
Model 2	1	1.00 (0.71–1.40)	1.40 (1.03–1.92)	1.49 (1.08–2.05)	0.002
Model 3	1	0.91 (0.65–1.28)	1.23 (0.90–1.67)	1.19 (0.85–1.62)	0.100
*CRP*					
Mean ± SD	0.41 ± 0.149	0.92 ± 0.170	1.81 ± 0.380	6.44 ± 6.714	
Incidence of diabetes *n* (*n*/1000)^a^	63 (3.7)	72 (4.6)	104 (6.4)	143 (9.3)	
Model 1	1	1.24 (0.89–1.74)	1.72 (1.26–2.36)	2.59 (1.93–3.49)	<0.001
Model 2	1	1.10 (0.78–1.56)	1.17 (0.84–1.64)	1.44 (1.03–2.00)	0.023
Model 3	1	1.24 (0.87–1.76)	1.28 (0.92–1.79)	1.40 (1.01–1.95)	0.046

Model 1: Adjusted for age and sex

Model 2: Adjusted for age, sex, waist circumference, smoking, use of anti-hypertensive medication, systolic BP, HDL, low educational level, and low physical activity at baseline

Model 3: Adjusted for age, sex, waist circumference, smoking, use of anti-hypertensive medication, systolic BP, HDL, low educational level, low physical activity, and fasting glucose at baseline

^a^Number of cases (incidence per 1000 person-years)

There was a significant interaction between orosomucoid and waist (*p* = 0.009) when checked for effect modification, indicating a lower effect of orosomucoid in subjects with high waist circumference. No significant interaction was observed between the other acute-phase proteins, and age, sex, and waist in the adjusted model (Model 2).

## Discussion

Low-grade inflammation has been associated with the incidence of diabetes [4–10]. However, the results from previous studies have been inconsistent and different for different inflammatory markers. The present study revealed significant associations between the acute-phase proteins orosomucoid, haptoglobin, and CRP and increased incidence of diabetes when adjusted for several potential confounders. However, after additional adjustment for fasting glucose, only CRP remained significantly associated with incident diabetes.

The results are in accordance with a study from the Malmö Preventive Project, which reported nonsignificant relationships for ceruloplasmin, alpha-1-antitrypsin, haptoglobin and orosomucoid and incident diabetes after adjustments for fasting glucose and other risk factors [22]. The association of orosomucoid seen in our study is in contrast to the findings of Schmidt et al. [11], who found an association between orosomucoid and diabetes after adjustment for several potential confounders including glucose. Similar to our study, no relation was found for alpha-1-antitrypsin and haptoglobin with diabetes. However, we found cross-sectional significant correlations between glucose and haptoglobin and orosomucoid, respectively. This is in accordance with findings from a case-control study by McMillan, which showed increased levels of haptoglobin, orosomucoid, and CRP in association with diabetes and glucose intolerance [6]. Ceruloplasmin and alpha-1-antitrypsin were less markedly raised in participants with diabetes [6].

Many studies have shown association between CRP and type 2 diabetes [6]. A prospective analysis and meta-analysis conducted by Lee et al. [23] showed that there is evidence of association between CRP and incidence of diabetes. However, these results were explained by central adiposity and markers of liver dysfunction. The meta-analysis demonstrated an overall relationship between CRP and diabetes, but there was a substantial heterogeneity in the results between studies, which reduced the degree of evidence in the meta-analysis [23]. A retrospective study conducted in a Japanese health screening population, where obesity was not prevalent, showed that hs-CRP, but not WBC, was independently associated with incident diabetes [24]. In the present population-based study, the results were controlled for confounders including waist circumference and baseline glucose levels and showed significant association in results.

It is widely accepted that systemic low-grade inflammation is associated with diabetes. However, inflammation is a heterogeneous concept, and the association between different inflammatory markers is often moderate. Interleukin (IL)-6, IL-1beta, and tumor necrosis factor-alpha (TNF-α) are important regulators of the hepatic production of acute-phase proteins [25, 26], but the regulation of the acute-phase protein production is very complex. Different cytokines operate in a network which results in additive, inhibitory, or synergistic regulatory effects of the production of different acute-phase proteins, depending on presence or absence of other cytokines and transcription factors, such as STAT3 or NF-κB [25]. Previous studies regarding the association between inflammation and incidence of diabetes have reported varying results for different inflammatory markers [5–9]. The varying relationships with incidence of diabetes could hypothetically be explained by differences with respect to the cytokines and transcription factors that regulate their production.

Dandona et al. [27] suggested two possible mechanisms involved in the pathogenesis of inflammation, and its link to obesity and diabetes. They proposed that firstly, chronic over-nutrition (causing obesity) can lead to a state of oxidative stress and hence inflammatory changes. Secondly, increased concentration of the pro-inflammatory cytokines such as TNF-α and IL-6 associated with obesity and diabetes might impair insulin action by suppressing insulin signal transduction [27]. The link between pro-inflammatory cytokines and insulin action was demonstrated by Hotamisligil et al., who showed increased expression of TNF-α in adipose tissue of obese rodents. A significantly increased peripheral uptake of glucose was observed in response to insulin when TNF-α was neutralized in obese fa/fa rats [28]. It was further demonstrated that TNF-α induces insulin resistance though an inhibitory form of insulin receptor substrate 1 [29]. Dandona et al. [30] established the association of higher serum TNF-α concentrations with obesity and significant fall in the levels with weight loss. In addition, it has been shown that a single high-fat high-calorie meal results in inflammation with increased expression of NFkB and many pro-inflammatory cytokines, including TNF-α [31, 32] which suggests that dietary factors could be a common link between inflammation and diabetes.

Many studies have shown associations between obesity, acute-phase proteins and diabetes [33, 34]. It can be argued that the link between inflammation and diabetes can be explained by the presence of obesity and adipose tissue which itself is a source of low-grade inflammation. However, this study has taken this fact into consideration, and the analysis has been adjusted for waist circumference. As the association is still significant, it can be speculated that there is a potential mechanism directly relating diabetes and inflammation. It should, however, be considered that previously it had been demonstrated that the association between CRP and diabetes is more likely to be non-casual, shown in the study by Brunner et al. [35]. Mendelian randomization was used in the study to demonstrate that the risk of developing diabetes was not associated with CRP levels in the European population. However, Brunner et al. [35] did not rule out the fact that acute-phase proteins early in the inflammatory process might be related to the development of diabetes.

### Strengths and limitations

The data used for this study is derived from a large-scale population-based cohort with a participation rate of 41 % [14]. The low participation rate stresses the importance to highlight the differences between the participants and non-participants in order to assess the external validity of the findings. Non-participants showed higher mortality than participants during and after the recruitment period, as well as during the follow-up. The participants in the MDCS were also compared to participants who took part in a mailed health survey (participation rate 75 %) in the same population. The participants of the cohort study showed comparatively better health but were still representative of the population in terms of socioeconomic structure, smoking habits, and obesity [14].

One advantage of the prospective design is that it enables the collection of information on exposure variables before onset of disease, which decreases potential recall bias and risk of reverse causation. The large sample size and long follow-up period enable detection of a large number of endpoint cases. Moreover, information about endpoints for this study was retrieved from various national registers and several data sources as mentioned earlier and has high validity [36]. Although multiple sources were used to ensure detection of all endpoint cases, it should be taken into account that diabetes can go undetected for a long time, and participants who did not seek medical attention might have been missed.

Some methodological issues need to be considered. Since this is an observational study, confounding is a main concern. Adequate adjustment for probable confounders was carried out in the Cox regression models. Although the analysis was adjusted for several possible confounders, the possibility of residual confounding cannot be ruled out.

Another limitation of the study is that the plasma levels of the acute-phase proteins were measured only at the baseline. The levels may have changed over time before the incidence of the disease. Similarly, anthropometric measurements were also carried out during the baseline visits and might have changed over the study period. One more limitation of this study is regarding the information on the type of diabetes. As the study cohort consists of middle-aged subjects, it can be assumed that almost all cases developed type 2 diabetes, as type 1 diabetes most often manifests at a younger age, and therefore were excluded as prevalent diabetes at baseline [37]. Another point to consider is that the cohort included participants being residents of Malmö, Sweden, only. However, based on previous reports by McMillan [6], Schmidt et al. [11], and Lee [23], it seems likely that the results can be generalized to other populations as well.

## Conclusion

In conclusion, this study demonstrated that there are associations between orosomucoid, haptoglobin, and CRP and the risk of incident diabetes. However, after additional adjustment for glucose, the association stayed significant for CRP. Further research in this regard is required to elucidate the underlying mechanism.

## Figures and Tables

**Fig. 4:**
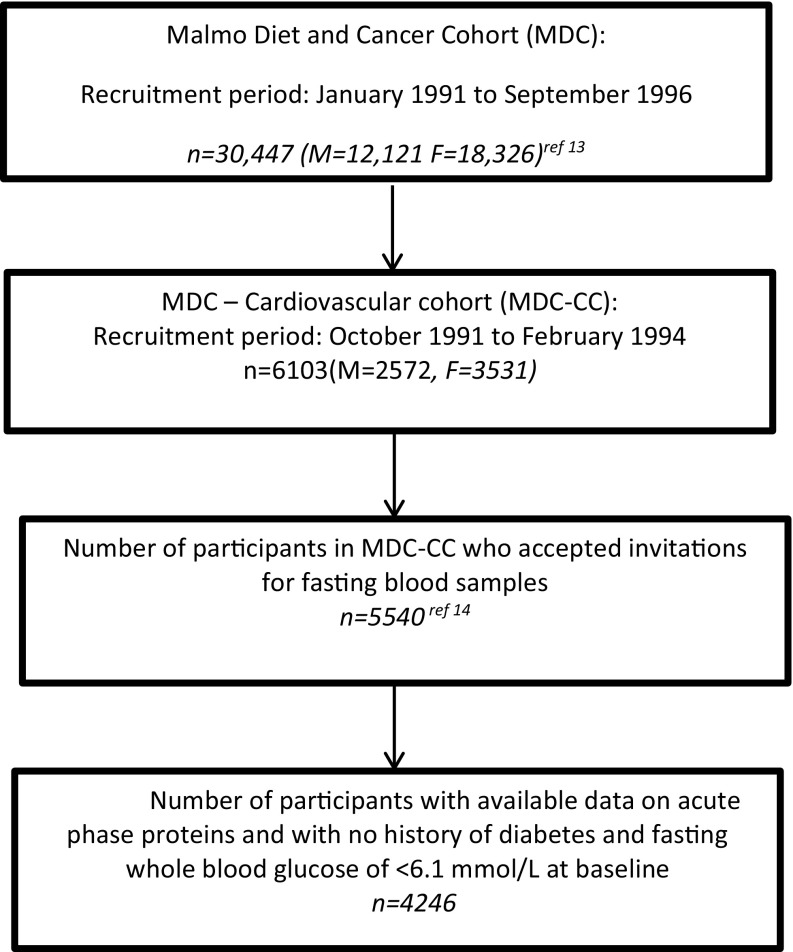
Flow chart

## Appendix

See Fig. 4.
